# Magnitude and determinants of treatment outcome among surgically treated patients with intestinal obstruction at Public Hospitals of Wolayita Zone, Southern Ethiopia: a cross sectional study, 2021

**DOI:** 10.1186/s12893-022-01568-1

**Published:** 2022-03-30

**Authors:** Muhaba Batebo, Bereket Loriso, Tilahun Beyene, Yosef Haile, Samuel Hailegebreal

**Affiliations:** 1 Homecho Primary Hospital, Gibe District, Southern Ethiopia, Ethiopia; 2 College of Medicine and Health Sciences, School of Medicine, Wolayita Sodo University, Wolayita Sodo, Ethiopia; 3 College of Medicine and Health Sciences, Wachamo University, School of Public Health, Hossana, Ethiopia; 4 College of Medicine and Health Sciences, Arba Minch University, School of Public Health, Arba Minch, Ethiopia; 5 Department of Health Informatics, College of Medicine and Health Science, School of Public Health, Arba Minch University, Arba Minch, Ethiopia

**Keywords:** Surgical outcome, Intestinal obstruction, Wolayita Zone

## Abstract

**Background:**

Procedures to treat intestinal obstruction range from minimally invasive laparoscopic surgery to more complicated open surgical procedures. It may end with high morbidity and mortality because of different reasons. It is very important to know about the determinants of favorable outcome of surgical management for intestinal obstruction however, little is known about this problem at public hospitals of Southern Ethiopia.

**Methods:**

Facility based cross sectional study was conducted. A total of 230 medical records which fulfill the inclusion criteria were used for this study. Variables with p value of less than 0.25 in the bivariate analysis were entered in multivariable logistic regression to control confounding. Finally, odds ratio with 95% confidence interval was used to identify variables which were significantly associated with dependent variable.

**Results:**

According to this study the magnitude of favorable surgical management outcome of intestinal obstruction was 177(77.0%) [95% CI, 71.4, 82.4]. Having small bowel obstruction (AOR = 2.49) [95% CI 1.91, 5.12], having simple bowel obstruction (AOR = 4.32) [95% CI, 2.00, 9.35], early presentation of patients (AOR = 4.44) [95% CI, 1.99, 9.92] and intraoperative procedure other than resection and anastomosis was performed (AOR = 0.45) [95% CI, 0.21, 0.96] were significantly associated with favorable outcome among surgically treated patients.

**Conclusion:**

The overall magnitude of favorable surgical management outcome of intestinal obstruction was moderate compared to other study. Having small bowel obstruction, having simple bowel obstruction, other procedure other than resection and anastomosis done, and early presentation of patients were significant predictors. Physicians should diagnose intestinal obstruction early and appropriate interventions should be taken on time before the complication happened. On time consultation and decision at the hospital setting is also recommended.

## Introduction

Intestinal obstruction is a gastro-intestinal condition in which digested material is prevented from passing normally through the bowel. It is inability of forward propulsion of intestinal content and the second causes of acute abdomen next to acute appendicitis [1–3]^.^

Globally, intestinal obstruction accounts for approximately 15% of all visits of emergency department for acute abdominal pain. The occurrence and etiology of bowel obstruction throughout the world varied depending on ethnicity, age group, dietary habits, and geographic location, among other factors [4, 5]. In the United States, 5% of all large bowel obstructions (LBOs) are caused by colonic volvulus, making it the third most common cause of LBO after cancer and diverticular disease in adult patients. In this country over 300,000 patients are estimated to undergo surgery to treat adhesion-induced small bowel obstruction annually. In some developing countries, including India, Pakistan and Brazil, sigmoid volvulus alone has been reported to account for 20% to 30% of all intestinal obstructions [6]^.^

According to different study, colorectal cancer is responsible for 60–70% of all large bowel obstructions, while diverticulitis and volvulus account for the majority of the remaining 30% worldwide. Although possible, abdominal wall hernias and adhesions rarely cause a true, isolated large bowel obstruction. In contrast, small bowel obstruction in most advanced western societies is caused most commonly by adhesions, neoplasms, or abdominal wall hernias. In Ethiopia sigmoid volvulas accounts for over 54% of intestinal obstructions [6, 7].

Studies in Attat and Ayder Referral Hospital, Mekelle in Ethiopia reported that the magnitude of intestinal obstruction accounted for 30 and 42% of all emergency surgically treated patients, respectively [8, 9]. A cross sectional study conducted in Arbaminch general Hospital in southern part of Ethiopia also reported that the overall magnitude of intestinal obstruction was 40.60% from all emergency surgically treated patients [10].

It is one of the serious surgical emergencies and associated with high mortality and morbidity throughout the World. Its mortality rates range from up to 3% for simple obstructions to as much as 30% when there is vascular compromise or perforation of the obstructed bowel, depending on the clinical setting and other related or unrelated co morbidities. In addition, bowel obstruction is frequently a recurrent problem, adding to the overall morbidity of an operation or successful non operative management [6, 7].

Management e of intestinal obstruction was improved since the development of more sophisticated diagnostic tests and imaging machine, but still the condition remains major public health problem especially in developing countries [9, 11, 12]. It is the challenging problem which is determined by many patients ‘related and clinical related factors like surgical site infection, wound dehiscence, leakage, pneumonia and sepsis. Many of this unfavorable outcome could be minimized if the factors related with surgical treatment outcome of intestinal obstruction is predetermined and all the necessary action is taken to prevent the causes associated with it before and after the procedure [13, 14].

Surgical management is evidence based surgical treatment and a concept on which surgical health professional relied on the updated and well evidenced data to recommend surgical operation for the patients. Physicians are not only expected to perform evidence-based procedure but also, they must understand about the postoperative recovery, the impact of quality of procedure and expected functional outcome [14].

Though studies have been done to assess the prevalence and causes of intestinal obstruction in Ethiopia, the condition related with the magnitude and determinants of treatment outcome among surgically treated patients with intestinal obstruction is largely remain unstudied especially in southern part of Ethiopia. Hence, the aim of this study was to assess magnitude and determinants of treatment outcome among surgically treated patients with intestinal obstruction at public hospitals in Wolayita Zone, Southern Ethiopia.

## Methods and materials

### Study design, study area and period

Facility based cross sectional study was employed for this study and it was conducted only at Wolayita Sodo University Teaching and Referral Hospital since all cases of intestinal obstruction from other public primary hospitals in the zone have been referred to this referral hospital. It provides referral services for patients referred from neighboring zones. Regarding the staffs, the surgical department of the hospital has 10 general surgeons, 4 orthopedic surgeons, 1 neuro-surgeon, 1 urologist, 1 maxillofacial surgeon, 1 pathologist and 1 HEENT surgeon. In the hospital there are two operation room, two male surgical wards, 1 female surgical ward, 1 pediatric surgical ward, 2 male orthopedic surgical wards, 1 female orthopedic surgical ward and 1 pediatric orthopedic surgical ward which are serving patients with surgical cases. The estimated number of surgeries in the previous year was five thousand cases. The department has 57 beds in general surgical wards and 36 beds in orthopedics ward. The study was conducted from June 1 to August 30, 2021 [15].

### Source population

All Patients’ medical records with the diagnosis of intestinal obstruction and treated surgically at Wolayita Sodo University Teaching and Referral Hospital in the previous three years (Jan 1, 2018–Jan 30 2021) was the source population.

### Study population

All complete patient’s records with the diagnosis of Intestinal Obstruction and managed surgically at Wolayita Sodo University Teaching and Referral Hospital during the study period were study population.

### Inclusion and exclusion criteria

All Patients with any age group admitted with the diagnosis of Intestinal obstruction and treated surgically at Wolayita Sodo University Teaching and Referral Hospital during the study period were included. However, all patients with incomplete data and all patients with missed medical record were excluded.

### Sampling procedure

All medical records of surgically managed patients for intestinal obstruction and those which fulfilled the eligibility criteria were collected initially. However, only 230 medical records fulfilled eligibility criteria. All the medical records were used for data collection. Then, the data collectors collected data from all available and complete patients’ medical record by using data collection checklist.

### Data collection tool and technique

Checklist that was developed after reviewing different related literatures was used [8–10, 16–18]. The checklist had six parts such as socio-economic and demographic characteristics, pre-operative factors, intra-operative factors, and post-operative factors, type of obstruction and causative agents of intestinal obstruction. Data were collected by 5 Bachelor of Science holder nurses using the pre-tested checklist. Two supervisors who were qualified with master of public health were recruited. The data collectors and supervisors were trained on the data collection tool, approach to the interviews, details of interviewing techniques, respect, and maintaining the privacy and confidentiality.

### Study variable

Surgical management outcome is a dependent variable while independent variables were socio demographic variables, pre-operative factors such as presenting symptom and progression of symptoms, duration of illness from the onset of symptoms up to surgery,, comorbidity, preoperative care received, previous abdominal surgery, intraoperative diagnosis and procedure done, type of obstruction, and causative agent.

### Operational definition

#### Surgical management outcome of intestinal obstruction

The condition of the patient after surgery done for intestinal obstruction that was whether she/he had “favorable” or “unfavorable” outcome according to the retrospective secondary data extracted from their medical records.

#### Favorable outcome

If the patient was discharged alive and hadn’t any history of postoperative complications, it was considered as favorable surgical management outcome.

#### Unfavorable outcome

If the patient died or has one or more postoperative complications like dehiscence, surgical site infection, pneumonia, and shock**.**

#### Simple bowel obstruction

When there is no vascular compromise in the intestine and if it is intact otherwise gangrenous.

#### Duration of illness

Is from onset of illness to time of management decision.

#### Pre-operative diagnosis

Clinical diagnosis before surgery by using presenting symptoms and laboratory diagnosis.

#### Duration of hospital stay

Time of hospital stay for the patients after surgery performed.

### Data quality assurance

Data quality assurance was maintained by performing different measures. To ensure the quality of data two-day training for data collectors and supervisors was given on procedure before data collection regarding surgical outcome of intestinal obstruction, on each part of the tool and ethical consideration. In addition the supervisor/principal investigator checked the collected data on daily basis in order to maintain its accuracy and completeness. Furthermore data collection tool was pretested at Wachamo University Nigist Ellen Memorial Referral Hospital in Hossana using 5% of the total sample size. Following the pretest any weakness in the structuring of the research instruments was identified, the tool was improved in terms of their clarity, understandability and simplicity in collecting the data required for the study.

### Data processing and analysis

Data were entered into epidata version 7.1 then it was exported to SPSS version 25 for analysis. Descriptive statistics was carried out and frequencies, pie charts and tables were used to present the data. Bivariate and multivariable logistic analysis was conducted. Variables in the bivariate analysis with p-value < 0.25 were the candidates for multivariable logistic analysis. Model fitness was good according to Hosmer and Lemshow goodness of fit test (p = 0.58). Statistical significance was declared at p-value 0.05 with 95% confidence interval. The magnitude of association between dependent and independent variables was determined by odds ratio with 95% confidence interval. Finally, the result was presented in text, frequency, percentages, tabulation, and charts.

## Results

### Socio-demographic characteristics of study participants

In this study, from a total sample size, 230 study participants’ data were retrieved from their medical records. Around 93(40.4%) participants were in the age category of 15–40 followed by the age category 41–60 years. The mean and standard deviation of the respondents’ age were 32.47 and ± 18.85 years, respectively. From the study participants more than half 153(66.5%) were male. Around 203(88.3%) participants were rural residents and 77(33.5%) participants were farmer (Table 1).

**Table 1:** Socio-demographic characteristics of study participants at public hospitals of Wolayita Zone, Southern Ethiopia (n = 230)

Variables		Frequency	Percent
Age category of the respondents	< 5	37	16.1
	5–14	17	7.4
	15–40	93	40.4
	41–60	68	29.6
	> 60	15	6.5
Sex	Male	153	66.5
	Female	77	33.5
Marital status	Married	153	66.5
	Single	76	33.1
	Divorced	1	0.4
Respondent’s religion	Protestant	149	64.8
	Orthodox	64	27.8
	Catholic	12	5.2
	Muslim	5	2.2
Residence	Rural	203	88.3
	Urban	27	11.7
Occupational status	Student	43	18.7
	Government employed	3	1.3
	NGO employed	1	0.4
	Merchant	46	20.0
	Farmer	77	33.5
	Non productive	60	26.1

### Lifestyle characteristics of study participants

This study has shown that none of patients had history of ever tobacco use even if history of tobacco use in most of the study participants was unknown from their medical records, 13(5.7%) ever alcohol use, and a history of illicit drug use was unknown for all patients from their medical records (Table 2).

**Table 2:** Lifestyle Characteristics of study participants at public hospitals of Wolayita Zone, Southern Ethiopia (n = 230)

Variables		Frequency	Percent
Tobacco use	Ever	0	0
	Never	67	29.1
	Unknown	163	70.9
Alcohol use	Ever	13	5.7
	Never	67	29.1
	Unknown	150	65.2
Illicit drug use	Ever	0	0
	Never	66	28.7
	Unknown	164	71.3

### Clinical characteristics and causes of intestinal obstruction

The findings revealed that abdominal pain 214 (93%), vomiting 196 (85.2%), nausea 183 (79.6%), abdominal distension 185 (80.4%), fever 66 (28.7%) and failure to pass abdominal contents, such as feces and/or flatus 138 (60%) were the leading clinical symptoms among patients presenting with intestinal obstruction.

**Fig. 1:**
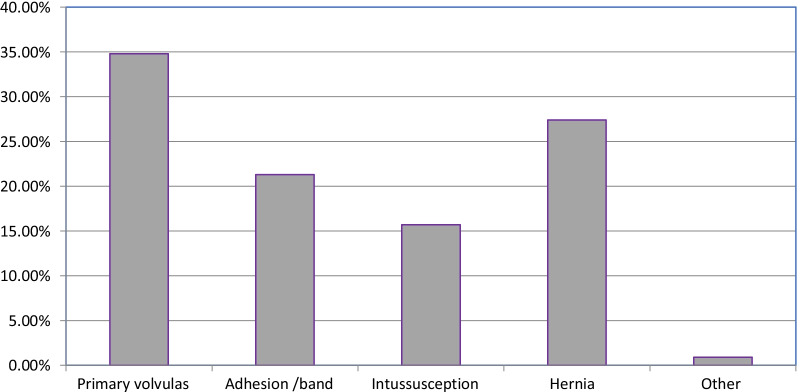
Causes of small bowel obstruction in the preoperative diagnosis of the study participants in Wolayita Sodo referral hospital, SNNPR Ethiopia

Concerning, the duration of illness or presentation of cases, 133 (57.8%) cases were presented longer than 24 h after the onset of symptoms of intestinal obstruction. Regarding, preoperative clinical diagnosis of the patients, small bowel obstruction accounts for 189 (82.2%). From the total intestinal obstruction 180(78.3%) were simple obstruction the remaining are gangrenous. Regarding the causes of small bowel obstruction primary volvulus was the leading causes 60 (31%). Others including adhesion, hernia and intussusception were the less common causes of SBO in the preoperative clinical diagnoses (Fig. 1).

### Intra-operative clinical characteristics

**Fig. 2:**
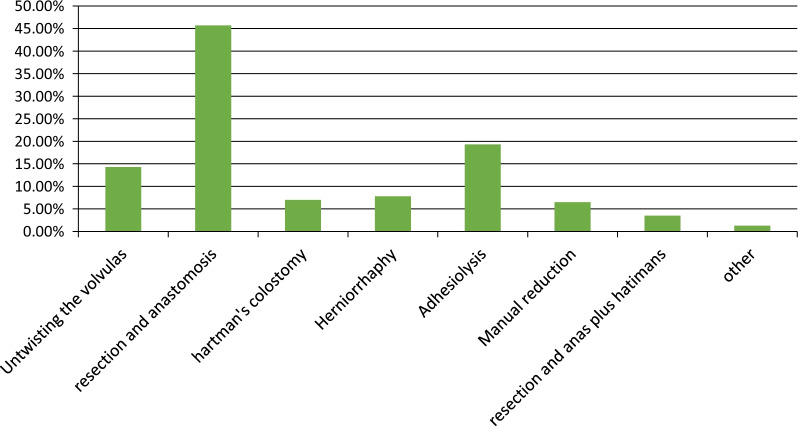
Intraoperative procedure done for the study participants in Wolayita Sodo referral hospital, SNNPR Ethiopia

Intussusception was the leading intraoperative clinical diagnosis of intestinal obstruction followed by simple sigmoid volvulus (SSV), gangrenous small bowel volvulus (GSBV), adhesion and band, strangulated hernia, incarcerated hernia, tumor and cancer, and gangrenous sigmoid volvulus (SBV) among others (Table 3). The commonest specific type of intraoperative procedure done after a general laparotomy was resection and anastomosis (45.7%) (Fig. 2).

**Table 3:** Intra-operative clinical diagnosis of the study participants at public hospitals of Wolayita Zone, Southern Ethiopia (n = 230)

Intraoperative diagnosis	Frequency	Percent
Simple sigmoid volvulas	33	14.3
Strangulated hernia	20	8.7
Adhesion and band	35	15.2
Intussusception	52	22.6
Tumor and cancer	7	3.0
Gangrenous sigmoid volvulas	14	6.1
Simple small bowel volvulas	23	10.0
Gangrenous small bowel obstruction	16	7.0
Incarcerated hernia	18	7.8
Ileosigmoid knotting	9	3.9
Other	3	1.3

### Surgical management outcome

**Fig. 3:**
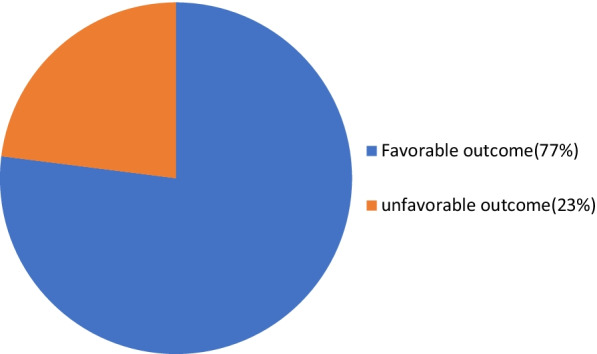
Surgical Management Outcome of the study participants in Wolayita Sodo referral hospital, SNNPR Ethiopia

The overall magnitude of favorable surgical management outcome of intestinal obstruction was found to be 177 (77.0%) [95% CI, 71.4, 82.4] (Fig. 3). From the total cases 18 (7.8%), 14 (7.4%), 6.1%, and 13 (5.7%) developed facial dehiscence, anatomic leak, septic shock and pneumonia at the postoperative period, respectively. The other complications are hypokalemia (3%), organ space surgical site infection 1(0.4%), superficial incision site infection 7(3%), and deep incision surgical site infection 10(4.3%). Two hundred ten (91.3%) of the cases of intestinal obstruction were improved at the discharge while 3.0% and 5.7% of cases were referred and died, respectively. From the total deaths most of the deaths (8 deaths) were reported from those complicated with septic shock. Furthermore 2 deaths were reported secondary to pneumonia and 2 deaths were also reported from cases complicated from facial dehiscence and anatomic leak.

### Factors associated with favorable surgical management outcome of intestinal obstruction

The variables associated with favorable surgical management outcome of intestinal obstruction in the bivariate logistic regression were co-morbidity, duration of illness/presentation of cases, type of obstruction (simple or gangrenous), Diagnosis (small or large bowel obstruction) and intraoperative procedure. Out of these five variables four variables were significantly associated with favorable surgical management outcome in multivariable logistic regression analysis. These were having small bowel obstruction, having simple bowel obstruction, other procedure other than resection and anastomosis done, and early presentation of cases.

Those having small bowel obstruction were about two times more likely to have favorable surgical management outcome than those who have large bowel obstruction (AOR = 2.49) [95% CI, 1.91, 5.12] and those who have simple bowel obstruction were about four times more likely to have favorable surgical management outcome than those who have gangrenous intestinal obstruction(AOR = 4.32) [95% CI, 2.00, 9.3].

In addition, those who came earlier within 24 h were about 4 times more likely to have favorable surgical outcome than those who came late to the hospital (AOR = 4.44) [95% CI, 1.99, 9.92]. Furthermore those for whom resection and anastomosis was performed were about 55% less likely to develop favorable outcome than the counterpart (AOR = 0.45) [95% CI, 21, 0.96] (Table 4).

**Table 4:** Multivariable Logistic Analysis for Factors Independently Associated with Surgical outcome of intestinal obstruction at public hospitals of Wolayita Zone, Southern Ethiopia (n = 230)

Variables	Category	Surgical outcome		COR with 95% CI	AOR with 95% CI	P value
		Favorable OC	Unfavorable OC			
Diagnosis	SBO	151 (20.1%)	38 (79.9%)	2.71 (2.51, 9.49)	2.49 (1.91, 5.12)	0.026
	LBO	15 (36.6%)	26 (63.4%)	1	1	
Type of obstruction	Simple	30 (15.9%)	159 (84.1%)	6.13 (3.26, 14.05)	4.32 (2.00, 9.35)	0.000
	Gangrenous	23 (56.1%)	18 (43.9%)	1	1	
Intraoperative procedure	Resection and anastomosis (yes)	72 (75.8%)	55 (25.2%)	0.29 (0.15, 0.57)	0.45 (0.21, 0.96)	0.041
	Resection and anastomosis (no)	90 (87.3%)	13 (22.7%)	1	1	
Duration of illness	Less or equal to 24 h	79 (86.8%)	12 (12.3%%)	4.98 (2.35, 10.3)	4.44 (1.99, 9.92)	0.000
	Greater than 24 h	98 (70.5%)	41 (29.5%)	1	1	

Where, 1 = Reference group, Hosmer and Lemeshow: p = 0.58 classification power = 80.0% Nagelkerke R square = 0.31

## Discussion

According to this study, moderate number of intestinal obstruction cases had favorable surgical management outcome from the total. The overall magnitude of favorable surgical management outcome of intestinal obstruction was 77.0% [95% CI, 71.4, 82.4]. This finding was in line with the finding of the study conducted in Harar (78.7%), Adama (75.4%) and India (74.11%) [10, 19, 20]. Even though this finding was higher compared with the studies conducted in eastern Ethiopia in Gelemso (67.2%), and Nigeria (33.5%) [21, 22], it was lower than the study conducted in Kenya (86.4%) [23]. The possible explanation for the above discrepancies might be due to variation in the distribution of the clinical and socio-demographic characteristics including place of residence of the study participants, the overall infrastructures of the study area and the hospital internal setups itself, the knowledge and skill of the health professionals regarding the diagnosis and management of intestinal obstruction.

This study revealed that having small bowel obstruction was one of the factors affecting surgical management outcome of intestinal obstruction. Those having small bowel obstruction were more likely to have favorable surgical management outcome than those who have large bowel obstruction. This could be due to the fact that the fast-healing nature because of high blood supply in small bowel as well as there is high bacterial load in large intestine. This finding was supported the study conducted in Harar [4].

In addition, those having simple bowel obstruction were more likely to have favorable surgical management outcome than those who have gangrenous bowel obstruction. This could be it is obvious that the intestine is intact in simple bowel obstruction but in gangrenous obstruction there is resection part which could increase poor outcome. As well as in gangrenous obstruction the load of bacteria is high which could increase the probability of septic infection. This finding consistent with the study conducted in Harar [4].

Furthermore, for those intraoperative procedures other than resection and anastomosis were performed were more likely to have favorable outcome than the counterpart. The possible explanation for this could be when resection and anastomosis is performed it makes wound on the bowel which can increases the chance of complications like paralytic ileus, anastomotic leak and early post operation adhesion. This finding was in line with the study conducted Adama [19]^.^

Patients who came to the hospital within 24 h were more likely to develop favorable outcomes than patients who came after 24 h. This was consistent with studies conducted in Gondar and Adama [19, 24]. This might be due to those who came early to the hospital have low chance of developing complication like sepsis, peritonitis, and the chance of developing gangrenous intestinal obstruction among those patients is also low. In addition, early and on time intervention for the patients increases the chance of favorability or early presentation in the case of intestinal obstruction decreases disastrous outcomes, notably a high rate of complications, long hospital stays, and high mortality.

### Limitation of the study

Since the study was cross sectional study it is difficult to predict cause and effect relationship. This study included small number of sample size since most of the medical records of patients were incomplete. Factors related with physicians skill and knowledge gap were not identified by this specific study since they have great importance on determining the outcome of surgically treated patients. The outcome or dependent variable of this study was chosen randomly which made less meaningful than treating each outcome separately.

## Conclusion

According to this study the overall magnitude of favorable surgical management outcome of IO in this study was moderate as compared to other study finding. Having small bowel obstruction, having simple bowel obstruction, other procedure other than resection and anastomosis done, and early presentation of cases were identified as factors which were significantly associated with favorable surgical management outcome of intestinal obstruction positively. Physicians should diagnose intestinal obstruction early and appropriate interventions should be taken on time before the complication happened. On time consultation and decision to refer at the primary hospital setting is also recommended. The health department of Wolayita Zone should improve public awareness on intestinal obstruction through health education and they should also improve referral linkage at lower-level heath institution for early referral. Starting from the health center the patient referral system should not be bad because it can increase the chance of complications.

## Data Availability

All documentary data and literature relevant to the study are publicly available. Interview data will not be shared to maintain confidentiality.
